# Sharing country experiences: The WHO Global School on Refugee and Migrant Health in Jordan

**DOI:** 10.3389/fpubh.2022.998920

**Published:** 2022-09-22

**Authors:** Saverio Bellizzi, Miriam Orcutt, Giuseppe D. Annunziata, Ana C. Sedas, Santino Severoni

**Affiliations:** ^1^World Health Organization, Amman, Jordan; ^2^World Health Organization, Geneva, Switzerland

**Keywords:** refugee, migrant, universal health coverage, Jordan, World Health Organization (WHO)

## Abstract

In 2021, Jordan was the first country to host the Global School for Refugee and Migrant Health, to improve the knowledge of the public health implications of migration. These perspective articles aim to retrieve salient reflections during the School as a baseline for further enhancement of migrant and health programs. During the School, a compilation of achievements, challenges, and opportunities was discussed around specific interrelated subjects, such as health system management and mental health. Successful examples were provided in the integration of refugees and migrants into health policies. On the other hand, the national health information systems are often not migrant-sensitive and evidence is still poor around mental health problems of refugees and migrants. Health financing remains a critical subject to address in a tailored way. The School highlighted the need to continue the exchange of experiences to promote a common approach to tackle similar needs.

## Introduction

Migration has become a structural phenomenon of the twenty-first century and^1^, in line with the vision supported by the Member States in May 2016 during the 69th World Health Assembly (WHA) (1), the right to health of all individuals must be considered in the design and implementation of programs at global, regional, national, and sub-national levels. The COVID-19 crisis has further exposed the high risks in terms of morbidity and mortality experienced by refugees and migrants due to inequities in access to health services (2). Lockdown and other measures taken by governments have additionally worsened the already precarious situation of many refugees and migrants, leading to income loss and additional healthcare insecurity (3). Inclusive policies can help refugees and migrants to maintain wellbeing, including good mental health, and healthcare systems can improve access to and engagement with the healthcare of host communities (4). The WHO has been working on managing the public health aspects of migration for the last decade. Specifically, WHO conducted several country assessments to understand the national health systems' capacity to manage large influxes of refugee migrants in the past years. One of the major findings was the need to increase the capacity and competence to properly address the health needs and rights of migrants and refugees in the hosting countries.

To note how WHO-EURO operated its first Summer School on Refugee and Migrant Health in Italy in July 2017, to improve participants' knowledge and understanding of the broader public health and health-system implications of large-scale migration in origin, transit, and destination countries (5).

In the same spirit, the newly established Health and Migration Programme in WHO Geneva has launched the Global School for Refugee and Migrant Health, which aimed to reach a diverse audience to strengthen understanding and knowledge to manage health systems and public health aspects of refugee and migrant health. This is also a key initiative to build capacity at the country and regional levels on migration and health.

In 2021, the Hashemite Kingdom of Jordan was the first country to host the Global School, with the online participation of more than 2,000 individuals across the globe. This School is built on the UN Sustainable Development Goals principle of “leave no one behind,” and the realization of universal health care for migrants and refugees based on evidence and on inclusive policies that balance the costs and benefits of “health for all” in a public health and development perspective (6).

The five-day course covered various subjects, such as refugee- and migrant-sensitive health systems and programs to include the health needs and rights of refugees and migrants in all aspects of the health services, including financing, policy, planning, implementation, and evaluation. The course curriculum was shaped in light of the participants' feedback and lessons learned in the past years; thus, converging with user demand in terms of quality and satisfaction. Key speech notes, subject-focused presentations, and panel discussions were alternated in addition to case-study videos from different settings to keep a balance between lectures and practical examples.

More importantly, the Global School attempted to provide a platform for input and discussion, as well as sharing of ground experiences and good practices from different regions of the world, with the participation of policymakers and health sector managers. Achievements, challenges, and opportunities were taken into consideration when sharing experiences around specific interrelated subjects, such as health system management, including financing and health information, and the impact of COVID-19 with a key focus on mental health.

The purpose of this piece is to retrieve the most salient reflections around the above-mentioned topics as a baseline for further enhancement and adjustment of migrant and health programs and policies.

## Health system

Sharing of good practices in the health systems in response to the migration of countries, such as Jordan, Guatemala, Bangladesh, Uganda, and Serbia, occurred. Successful examples were provided on the integration of refugees and migrants in health policies, such as universal access to treatment and vaccination for COVID-19. On the other hand, several nuances exist, which relate to various obstacles to healthcare, especially legal status, discrimination, and fear. One of the main challenges that emerged was the fact that migrants are often shouldered by countries with overburdened health systems and often suboptimal health coverage for the local population.

The Rohingya situation in Bangladesh represents a striking example, with more than one million refugees accommodated in a low-income country, where universal quality access to healthcare is also an issue for the local population (7). Similarly, migrants and refugees in large parts of Africa face increasing difficulties to access food in addition to medicines, which has prompted the civil societies in countries like Nigeria to make efforts at the House of Representatives for some budgetary provisions for the healthcare of IDPs, migrants, and refugees ^2^. Guatemala experience further elucidation of the dire urgency needed to deal with the reality of the migrant crisis in Central America, particularly the Northern Triangle ^3^.

## Health information

A key point raised during the week was about accurate health information. Specifically, population-based surveys and national health information systems are often not migrant sensitive, which highlights the need for harmonized information tools to enable planning and better preparedness. Promising solutions were presented, such as the Support Asylum and Vulnerabilities, through the e-health (SAVe) experience by the Italian National Institute for Health, Migration, and Poverty (INMP). This would allow an accurate illustration of the clinical status of migrants concerning privacy. The interactive session expressed the need to expand digital health records across borders to ensure proper diagnostics and a continuum of care from countries of origin to countries of transit and destination. This is particularly important in times of epidemiological transition, with many migrants and refugees affected by non-communicable diseases, such as diabetes and hypertension. Additionally, developing universal e-solutions (i.e., e-medical cards) for refugees to track health care provision and immunization was suggested to track the healthcare, given the living conditions of refugees who are on the move, to note how this reflects the concept of essential health services that emerged during the COVID-19 pandemic.

## Mental health

In addition to the steady emerging burden of non-communicable diseases, particular attention was dedicated to mental health and health promotion; the latter is critical to cover a wide range of social and environmental interventions that are essential to properly address the health needs and rights of refugees and migrants within the framework of inclusive good governance for health. While the current view on the mental health burden among refugees and migrants was reported by both IOM and WHO specialists, it is worth noticing that evidence is still poor and more bodies of research regarding the mental health problems of refugees, migrants, and IDPs are needed. Specifically, mental health status is the result of complex interactions, again, related to departure/transit/arrival. It is also closely connected to the legal status, discrimination, communication with host populations, and other relevant factors.

## Health financing

Probably, one of the most important aspects related to achieving UHC, with the inclusion of refugees and migrants, is still health financing. The interactive part of the last day of the Global School emphasized how health financing in sub-Saharan Africa (SSA) is often minimally supported by national health insurance, with external donor funding and out-of-pocket payments accounting for one-third of each of the total. In parallel, when expanding health access and services to residents and migrants in areas with limited finance for healthcare, there should be a prioritization in terms of burden, severity, and costs of services, with a specific focus on primary healthcare services (primary package of health services). In this regard, a “basket of benefits” is a crucial parameter in the health coverage of migrants anywhere in the world, and there is a question on which package of benefits would be more suited to migrants. On the other hand, a balanced approach is probably needed by the time the health insurance system coverage grows larger in a country (which provides a good range of coverage), and the same country becomes a preferred destination for immigrants (because of free access to healthcare).

## Conclusions

The 2021 edition of the WHO Global School for Refugee and Migrant Health convened more than 2,000 participants from all regions of the world, who converged on the concept that refugees and migrants should be considered as an active component of the community landscapes. Hence, tailored strategies to optimize access to quality health care are functional to maximize the contribution of such components to the overall society.

Specifically, a country's experiences highlight the need to continue the exchange of experiences by various countries to promote a common approach, keeping, of course, in mind the variety of contexts but identifying similarities and common needs.

There is a need for example, for a global fund to support countries, such as the Global Fund, coordinated by philanthropic institutions as well as institutions like the World Bank, supported by international organizations, such as WHO, IOM, UNHCR, and others. Indeed, contextualization is needed to tailor an adequate response by different national health systems. This will be based on local epidemiology, specific vulnerabilities, as well as available resources, and clear planning, considering health literacy and language barriers. As highlighted by some interventions during the first day, universal health coverage programs in the African context remain not fully matched for several reasons, including the frequent public health emergencies affecting the regions that are absorbing the existing resources, and the financial crisis due to the COVID-19 pandemic. However, refugee and migrant health needs and rights are not fully addressed in any part of the globe.

Rethinking is a must to allow sustainable mechanisms for the country's financing with political commitment, supported by technical and financial partners.

The impact of the course was measured through short questionnaires released during and after the five days of the School, which revealed an appreciation for the formula used for the 2021 Global School. To note how the hybrid format used for the Global School in Amman allowed for particularly efficient use of resources. In terms of human resources, a core group of staff from WHO HQ, WHO EMRO, and WHO Jordan (around 10 people in total) was devoted to the preparation and conduction of the event. The event cost <50,000 USD, which resulted in <50 USD cost per capita when compared with previous editions hosted in person that cost more than 2,500 USD per capita.

## Data availability statement

The original contributions presented in the study are included in the article/supplementary material, further inquiries can be directed to the corresponding author.

## Author contributions

SB and MO produced the initial outline and conceptualized the manuscript. All other authors contributed to the manuscript in writing and review of content and have approved it for publication.

## Conflict of interest

The author declares that the research was conducted in the absence of any commercial or financial relationships that could be construed as a potential conflict of interest.

## Publisher's note

All claims expressed in this article are solely those of the authors and do not necessarily represent those of their affiliated organizations, or those of the publisher, the editors and the reviewers. Any product that may be evaluated in this article, or claim that may be made by its manufacturer, is not guaranteed or endorsed by the publisher.

## Footnotes

^1^ https://www.who.int/health-topics/refugee-and-migrant-health#tab=tab_2

^2^ https://www.acaps.org/country/nigeria/crisis/cameroonian-refugees

^3^ https://www.msf.org/central-american-migration-depth
